# Effect of the pulpal hydrostatic pressure on the morphological data of the fluid droplets emerging from dental enamel in human teeth

**DOI:** 10.1016/j.dib.2020.105901

**Published:** 2020-06-20

**Authors:** Ekachai Chunhacheevachaloke, Ratirach Tanapitchpong, Orapin Ajcharanukul

**Affiliations:** a Department of Pediatric and Preventive Dentistry, Srinakharinwirot University, Bangkok 10110, Thailand; b Department of Stomatology, Faculty of Dentistry, Srinakharinwirot University, 114 Sukhumvit 23, Bangkok 10110, Thailand

**Keywords:** Dental enamel, Bicuspid, Dentinal fluid, Hydrostatic pressure, Human, Tooth permeability

## Abstract

Enamel fluid in human teeth plays an important role in the permeability and mechanical property of the enamel. It can be invetigated as fluid droplets at the enamel surface by using the replica technique. The experiments were done on 17 premolars of 10 subjects (aged 10–25 yrs) to be extracted during orthodontic treatment. Teeth were divided into 2 groups. In the first group (*n* = 11), the fluid accumulated on the mid buccal of the enamel surface was recorded with the impression material both *in vivo* and *in vitro. In vitro* replicas were obtained when the pressure in the pulp cavity held at 0, 20, 100, and 200 mmHg above atmospheric. They were examined by a scanning electron microscope. For the other group (*n* = 10), each tooth was prepared for fluid flow measurement during which the same set of pulpal pressures was applied as mentioned in the other study [1]. However, four teeth of 3 subjects were arranged for the recording of fluid conductance, while the replica impression at the mid buccal surface was also taken simultaneously under each of the applied pulpal pressure. This dataset describes subjects’ baseline characteristics, including their corresponding records of the droplets’ observations both *in vivo* and *in vitro*. Also, data of *in vitro* fluid flow measurements were detailed according to the applied pulpal pressure. The patterns of changes of the fluid flow rates and the droplets’ numbers provided in this dataset can be used to validate tests of agents affecting the structure and permeability of the enamel.

**Specifications Table**

**Table:** 

**Subject**	Dentistry, Oral Surgery and Medicine
**Specific subject area**	Adhesive Dentistry, Oral Physiology, Orthodontic biology
**Type of data**	Tables
	Figures
**How data were acquired**	Scanning electron microscope (SEM), A digital microscopy software (Motic Image Plus version 2.0, Motic China Group CO., LTD., Fujian, China)
	Model for the recording of fluid flow through the crown [1]
**Data format**	Raw:
	-Tables 1, and 3
	-Figure 1
	-Figs. 2, and 3 were linked to Mendeley Data: http://dx.doi.org/10.17632/jcf95fckwh.1
	Filtered: Table 2
**Parameters for data collection**	Young adults’ premolars scheduled for extraction as a part of orthodontic treatment. The teeth were fully erupted, free from caries and restorations.
**Description of data collection**	Before tooth extraction, replicas were obtained in the mid buccal premolars using polyvinylsiloxane impression material. Positive replicas were then collected using a polyether impression material, and inspected by a SEM. *In vitro*, each tooth was prepared for fluid flow measurement during which the pressure in the pulp cavity was set at 0, 20, 100 and 200 mmHg above atmospheric. All replicas were obtained with each of the different pressures exactly the same procedure as that of *in vivo*.
**Data source location**	Srinakharinwirot University, Faculty of Dentistry
	Bangkok, Thailand
**Data accessibility**	With the article:;
	- Tables 1, 2, and 3
	- Figure 1
	Figures 2, and 3:
	-Repository name: Mendeley Data
	-Data identification number: 10.17632/jcf95fckwh.1
	-Direct URL to data: http://dx.doi.org/10.17632/jcf95fckwh.1
**Related research article**	Tanapitchpong, R., Chunhacheevachaloke, E., Ajcharanukul, O. *In vivo* and *in vitro* study of enamel fluid flow in human premolars, Archives of Oral Biology 117 (2020) 104795, http://doi.org/10.1016/j.archoralbio.2020.104795.

**Value of the Data**

The data provide proof of the relationship between the morphological observations of fluid droplets at the enamel surface and the hydraulic conductance through the tooth crown.
Dental practitioners, dental students, and researchers can benefit from an understanding of the fluid flowing through the enamel and its importance for the enamel permeability.
The model for recording the hydraulic conductance through the crown can be used to validate the efficacy of anti-caries agents, dental restorations, or dental adhesives.
The observations of fluid droplets at the enamel surface *in vivo* provide the information of the enamel surface of human premolars in young adults. They can be combined with others' datasets of either various teeth or age, and analyzed for further insights.

## 1 Data description

**Fig. 1:**
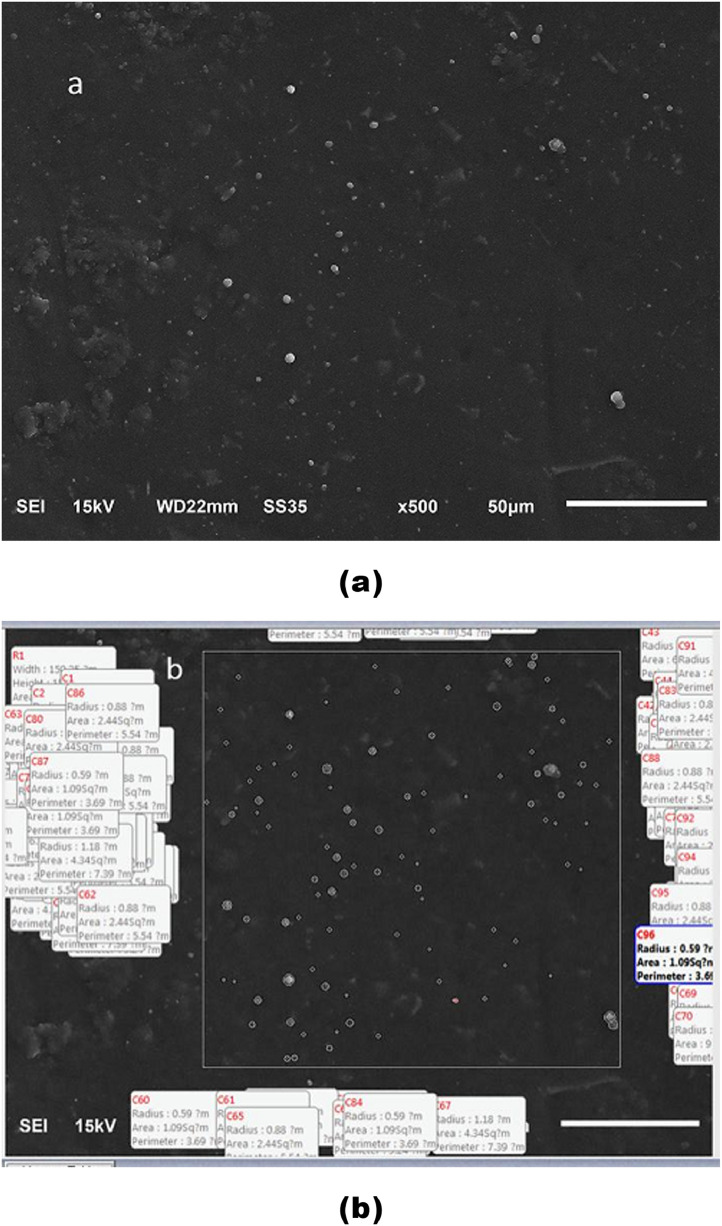
Scanning electron micrograph of replica of tooth 45 (Subject A) obtained after applying of +100 mm Hg *in vitro* (a), the number and size of droplets were determined using a digital microscopy software (b).

Baseline characteristics of the subjects such as age, gender, and tooth type were detailed according to the experimental procedures in Tables 1, 2, and 3. Raw data of the morphological observations describing the number of the droplets were obtained from 11 human premolars of 6 subjects both *in vivo* and *in vitro* (Table 1). The *in vitro* data perceived when the pressure in the pulp cavity was held at 0, 20, 100, and 200 mm Hg were then utilized for the regression analysis to find the relationship between the increased pulpal pressure and the change in the number of droplets [1]. Since droplets appeared in the SEM images varied in size, data of the droplets’ diameters obtained from each sample were calculated as mean value (Table 2). The method for numbering and sizing of the droplets by using a digital microscopy software was demonstrated in Fig. 1b. More sample images of the replicas obtained from two subjects under different conditions can be downloaded from the Mendeley Database (Figs. 2, and 3).

**Table 1:** Number of fluid droplets that appeared on the mid-buccal area of the tooth surface *in vivo* and *in vitro*.

Subject (gender)	Age	Tooth	Number of fluid droplets per 22,500 µm^2^				
			*In vivo*	Applied pulpal pressure *in vitro* (mm Hg)			
				0	20	100	200
W (F)	11	44	105	91	116	108	124
W (F)	11	45	60	76	117	93	155
X (F)	22	35	47	90	61	106	134
X (F)	22	24	77	104	106	92	87
Y (M)	25	25	60	52	58	75	110
Z (M)	25	35	88	56	89	90	104
A (M)	16	24	63	61	51	73	71
A (M)	16	35	38	47	63	78	90
A (M)	16	14	88	39	84	106	114
A (M)	16	45	73	30	89	96	111
B (M)	10	24	79	43	65	65	65

**Table 2:** Mean diameters (µm) of fluid droplets that appeared on the mid-buccal area of the tooth surface *in vivo* and *in vitro*.

Subject (gender)	Age	Tooth	Mean diameter (µm) of fluid droplets per 22,500 µm^2^				
			*In vivo*	Applied pulpal pressure *in vitro* (mm Hg)			
				0	20	100	200
W (F)	11	44	2.54	1.78	2.2	1.76	2.38
W (F)	11	45	1.89	1.78	2.1	2.14	1.86
X (F)	22	35	2.54	2.68	4.48	2.28	3.3
X (F)	22	24	1.98	2.32	2.12	2.14	1.82
Y (M)	25	25	1.7	2.34	3.04	2.66	2.7
Z (M)	25	35	1.84	1.78	2.54	2.7	2.76
A (M)	16	24	2.18	1.62	1.9	1.62	2
A (M)	16	35	1.84	1.8	1.88	1.64	1.74
A (M)	16	14	1.98	2.18	2.4	2.4	2.1
A (M)	16	45	1.62	1.7	2.1	2.06	2.36
B (M)	10	24	1.72	1.76	1.78	1.78	1.58

**Table 3:** Fluid flow rates through the crown recorded under different positive pulpal pressures.

Subject (gender)	Age	Tooth	Fluid flow rate (nL / min)			
			Applied pulpal pressure *in vitro* (mm Hg)			
			20	100	200	300
W (F)	11	44	101.0	212.1	424.3	606.1
W (F))	11	45	86.6	157.1	424.3	530.3
K (F)	17	24	65.3	192.8	471.4	606.1
K (F)	17	14	106.1	249.6	424.3	606.1
Z (M)	25	14	83.2	249.6	471.4	606.1
Z (M)	25	35	84.9	249.6	424.3	606.1
S (F)	24	34	65.3	265.2	385.7	606.1
V (F)	21	24	75.8	169.7	385.7	606.1
L (F)	25	14	68.4	184.5	326.4	606.1
X (F)	22	35	86.6	249.6	385.7	530.3

Dataset of fluid flow rates collected from 10 recently extracted premolars of 7 subjects using the model for the recording of fluid flow through the tooth crown [1] was shown in Table 3. However, 4 teeth of 3 subjects were arranged for the recording of fluid conductance, while the replica impression at the mid buccal surface was also taken simultaneously under each of the applied pulpal pressure (Tables 1, and 3). The data were used for the regression analysis and then further calculated for the hydraulic conductance of the tooth crown [1].

## 2 Experimental design, materials, and methods

### 2.1 Subjects

All procedures performed and all treatments in these studies involving human participants were conducted strictly in full accordance with the ethical principles and standards of the institutional and national research committee and with the 1964 World Medical Association Declaration of Helsinki and its later amendments (version 2008) or comparable ethical standards. Informed consent of the subject was obtained under the study protocol reviewed and approved by an ethics committee from the Faculty of Dentistry, Srinakharinwirot University.

### 2.2 Materials, and methods

The experiments were carried out on 17 healthy premolars of 10 subjects (aged, 10–25 yrs). The teeth were divided into two groups: replica impression (*n* = 11) and fluid flow measurement (*n* = 10) groups. In the replica impression group, the resin replicas were obtained from the mid-buccal of the enamel surface of 11 premolars both *in vivo* and *in vitro* using a slow setting polyvinyl siloxane impression material (Affinis light body; Coltene, Alstatten, Switzerland) that incorporates the exudation in droplet-like formations. Epoxy resin replicas obtained by using a polyether impression material (Permadyne Garant, 3 M ESPE, St. Paul, MN, USA) were produced from these impressions and observed under SEM (JEOL, Model 5400, Tokyo, Japan) [2,3]. After extraction, each tooth was sectioned transversely at the cemento-enamel junction with a diamond disk and water coolant. Any remaining coronal pulp tissue was removed with fine tweezers. The pulp chamber was then irrigated with water from a triple syringe for 10 min and filled with 2.5 N sodium hydroxide for 3 days to remove any remaining tissue and odontoblasts [4]. The *in vitro* experiment was conducted by using the model for fluid flow measurement [5]. After the pressure in the pulp cavity was set at 0, +20, +100, and +200 mm Hg, the replica was made from the enamel surface of the crown at 5 min following each of the applied pressure by using the same procedure as that *in vivo.* Digital microscopy software (Motic Image Plus version 2.0, Motic China Group CO., LTD., Fujian, China) was utilized to observe the number and diameter of droplets that appeared on each replica. The example of the morphological observation of the replica-SEM was demonstrated in Fig. 1. The SEM photomicrographs of replicas obtained from 2 subjects were shown in Figs. 2 and 3 (the Mendeley Database).

For the fluid flow measurement group, a recently extracted tooth was prepared as above. A similar setting for the measurement of the fluid flow rate through the tooth crown was performed following the applications of the same series of positive pulpal pressures [5]. However, four teeth of 3 subjects were arranged for the recording of fluid conductance, while the replica impression at the mid buccal surface was also taken simultaneously under each of the applied pulpal pressure.

The SigmaPlot 11.0 (Systat Software Inc., San Jose, CA, USA) was used to statistically analyze the differences among the experimental variables including the possible relationship between either change of the droplet's number or the fluid flow rate associated with the increasing pulpal pressure.

## Declaration of Competing Interest

The authors declare that there is no known competing financial interests or personal relationships which have, or could be perceived to have influenced the work reported in this article.
